# Deep Subseafloor Fungi as an Untapped Reservoir of Amphipathic Antimicrobial Compounds

**DOI:** 10.3390/md14030050

**Published:** 2016-03-10

**Authors:** Marion Navarri, Camille Jégou, Laurence Meslet-Cladière, Benjamin Brillet, Georges Barbier, Gaëtan Burgaud, Yannick Fleury

**Affiliations:** marion.navarri@univ-brest.frcamille.jegou@univ-brest.frlaurence.meslet@univ-brest.frbenjamin.brillet@univ-brest.frgeorges.barbier@univ-brest.frgaetan.burgaud@univ-brest.fr

**Keywords:** deep subseafloor fungi, antimicrobial metabolites, amphipathic compounds, solid-state fermentation

## Abstract

The evolving global threat of antimicrobial resistance requires a deep renewal of the antibiotic arsenal including the isolation and characterization of new drugs. Underexplored marine ecosystems may represent an untapped reservoir of novel bioactive molecules. Deep-sea fungi isolated from a record-depth sediment core of almost 2000 m below the seafloor were investigated for antimicrobial activities. This antimicrobial screening, using 16 microbial targets, revealed 33% of filamentous fungi synthesizing bioactive compounds with activities against pathogenic bacteria and fungi. Interestingly, occurrence of antimicrobial producing isolates was well correlated with the complexity of the habitat (in term of microbial richness), as higher antimicrobial activities were obtained at specific layers of the sediment core. It clearly highlights complex deep-sea habitats as chemical battlefields where synthesis of numerous bioactive compounds appears critical for microbial competition. The six most promising deep subseafloor fungal isolates were selected for the production and extraction of bioactive compounds. Depending on the fungal isolates, antimicrobial compounds were only biosynthesized in semi-liquid or solid-state conditions as no antimicrobial activities were ever detected using liquid fermentation. An exception was made for one fungal isolate, and the extraction procedure designed to extract amphipathic compounds was successful and highlighted the amphiphilic profile of the bioactive metabolites.

## 1. Introduction

The increasing trend of bacterial resistance currently jeopardizes global public health and thus the global healthcare system. Outbreaks due to antimicrobial resistant microorganisms kill about 3 million people each year in the world [1] including 23,000 American [2] and 37,000 European people [3]. The threat is of particularly of concern as most of the currently available antibiotics have lost their efficiency [4]. To counteract antibiotic resistance, Centers for Disease Prevention and Control are developing and financing research programs dedicated to (i) the prevention of healthcare-associated infections; (ii) the improvement of antibiotic prescription; and (iii) the promotion of new antibiotics exploration.

Natural and natural-derived drugs in pharmaceutical pipelines represent 71% of newly approved molecules [5]. According to J. Bérdy (2012), 60,000–80,000 microbial compounds have been characterized, 32,000–34,000 were bioactive of which 15,000 are of fungal origin [6] some of them being real blockbusters. Some examples of fungal success stories are the well-known (i) penicillin, which started the antibiotic era [7], statins preventing coronary heart diseases [8] or cyclosporins as immunosuppressive agents [9].

Marine ecosystems paid special attention as a new reservoir of antimicrobials as emphasized by Blunt *et al.* since 2003 in their annual reviews on marine natural products. Oceans harbor a broad diversity of ecosystems colonized by a huge diversity of microorganisms synthetizing a wide array of original bioactive metabolites [10,11]. Marine fungi have usually been considered as uncommon microorganisms and are still largely underexplored. Logically, the number of marine fungal natural products characterized to date (almost 700 new molecules between 2006 and 2010 [12]) remains low, albeit following an increasing trend. However, marine fungal chemodiversity appears original with numerous novel structures described. As numerous shallow-water marine fungi have been screened for bioactive compounds, the challenge now is to search for untapped fungal resources in untargeted ecological niches as marine extreme environments. Deep-sea ecosystems, as deep-sea hydrothermal vents or the deep subseafloor appear as extreme and complex habitats, *i.e.*, harboring numerous microorganisms [13], which may be allegorized as chemical battlefields [14] of interest for biotechnological applications.

In this underexplored context, a collection of almost 200 deep subseafloor fungal strains obtained from a sediment core, recently established by Rédou *et al.* [15], highlighted (i) the fungal diversity within the deep-subseafloor from 4 to 1884 mbsf and (ii) its biotechnological potential using genome mining for specific genes involved in biosynthesic pathways of bioactive secondary metabolites [16]. Here, we report the functional screening of this deep subseafloor fungal collection for antimicrobial activities. A set of 110 fungal strains was selected, as a representative sub-collection, in all identified clusters and at different depths of isolation. We also discuss their ecological significance and finally report first insights into the antimicrobial compound production and their chemical nature. 

## 2. Results and Discussion

### 2.1. Antimicrobial Screening

**Figure 1:**
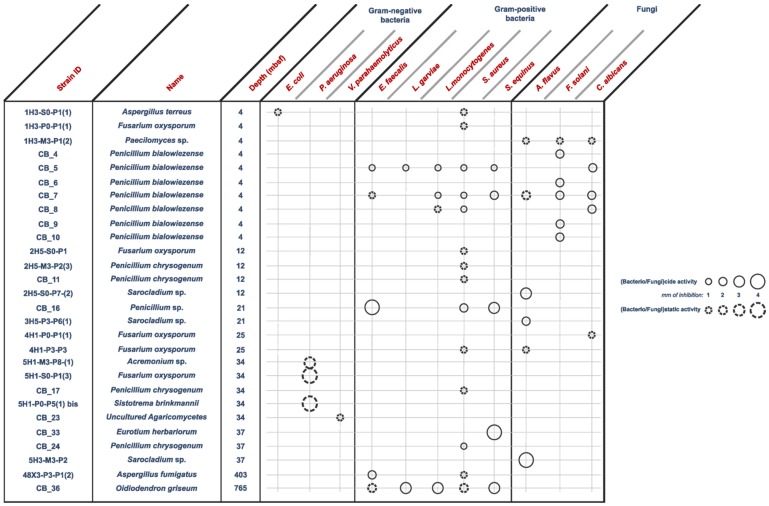
Antimicrobial spectrum of the 28 deep subseafloor antimicrobial producing fungal isolates.

A selection of 110 deep subseafloor fungal isolates was screened against a panel of 16 microbial targets using agar diffusion method. Surprisingly, in our culture conditions, no antimicrobial activity was ever detected from the 24 assayed marine yeast isolates, identified as strains of *Bullera alba*, *Meyerozyma guillermondii* and *Rhodotorula mucilaginosa* species [15]. Some terrestrial representatives of those species were already demonstrated as antimicrobial peptide producers [17,18,19,20]. Alternatively, a high proportion of filamentous fungi exhibiting antimicrobial activities was revealed. Indeed, 28 fungal strains of the 86 assayed exhibited antimicrobial activity (even partial) against at least one microbial target (Figure 1 and Figure S1). Thus, around 33% of the deep subseafloor filamentous fungal collection was shown to produce antimicrobial compounds. Such a high proportion of antimicrobial producing fungal strains has already been described from marine sediment- or invertebrate-associated fungi [21,22,23,24]. As a comparison, a screening of deep-sea surface sediment bacteria against eight microbial targets revealed 13% of bacterial strains synthesizing bioactive compounds [25]. Thus, deep-sea complex habitats, such as deep subseafloor, do appear as reservoirs of bioactive secondary metabolites.

Fungal strains synthesizing antimicrobial compounds could be divided into three groups depending on their antimicrobial spectrum:

- Anti Gram-positive fungi, as the most important group clustering 15 strains (53% of the bioactive strains). Isolates were identified as belonging to *Aspergillus fumigatus* 48X3-P3-P1(2), *Aspergillus terreus* 1H3-S0-P1(1), *Eurotium herbariorum* CB_33, *Fusarium oxysporum* (1H3-P0-P1(1), 4H1-P0-P1(1) and 4H1-P3-P3), *Penicillium bialowiezense* (CB_5, CB_7 and CB_8), *Penicillium chrysogenum* (2H5-M3-P2-(3), CB_11, CB_17 and CB_24), *Penicillium* sp. CB_16 (1 strain), and *Oidiodendron griseum* CB_36 species. Marine species of *Penicillium*, *Aspergillus* and *Fusarium* genera are well-known as producers of a wide array of bioactive metabolites, *i.e.*, polyketides, alkaloids and peptides, with a broad spectrum of biological activities [12,26,27,28]. Several marine-derived *Eurotium* species have been investigated for antimicrobial activity with a special focus on *Eurotium cristatum*. An algae-associated *E. cristatum* strain was assessed as producer of the tardioxopiperazine A antibiotic inhibiting *Staphylococcus aureus* [29] contrary to one sponge-associated *E. cristatum* strain [30]. Interestingly, and to the best of knowledge, no antimicrobial activity was ever detected from marine-derived *Oidiodendron* strains while, some terrestrial strains, as *Oidiodendron truncatum* or *Oidiodendron fuscum*, are able to synthesize antibacterial polyketide (fuscin) [31] and terpenoid (clerocidin) [32].
- Anti-fungal strains gathering: *Penicillium bialowiezense* (CB_4, CB_5, CB_6, CB_7, CB_8, CB_9 and CB_10), *Sarocladium* sp (2H5-S0-P7(2), 3H5-P3-P6(1) and 5H3-M3-P2), *Fusarium oxysporum* (4H1-P0-P1(1) and 4H1-P3-P3) and *Paecilomyces* sp. 1H3-M3-P1(2) species. Marine species of *Penicillium* and *Fusarium* genera have already been described as producers of antifungal terpenoids like penicisteroids and polyketides as fusarielin E, respectively [33,34,35,36,37,38]. Marine-derived *Paecilomyces* species, isolated from mangrove habitats, have been shown to produce antifungal polyketides [39,40]. *Sarocladium kiliense* and *Sarocladium oryzae* are known to produce antifungal terpenoid and polyketide against phytopathogenic fungi [41] and *C. albicans* [42,43]. A marine endophytic *Sarocladium* sp. was described for its ability to inhibit bacterial *quorum sensing* [44]. Here, we report an original ability of a deep subseafloor *Sarocladium* isolate to biosynthesize bioactive compounds against *A. flavus*.
- Five deep subseafloor fungal isolates had a weak antibacterial activity against Gram-negative bacteria (partial inhibition). Those isolates were identified as *Acremonium* sp. 5H1-M3-P8(1), *Aspergillus terreus* 1H3-S0-P1(1), *Fusarium oxysporum* 5H1-S0-P1(3), *Sistotrema brinkmanii* 5H1-P0-P5(1)bis and an uncultured Agaricomycete CB_23. If species of *Aspergillus*, *Fusarium* and *Acremonium* genera have already been described to produce an array of anti-Gram-negative compounds [45,46,47,48,49,50], to the best of knowledge we here described an original antimicrobial activity from a *Sistotrema brinkmannii* species.

**Figure 2:**
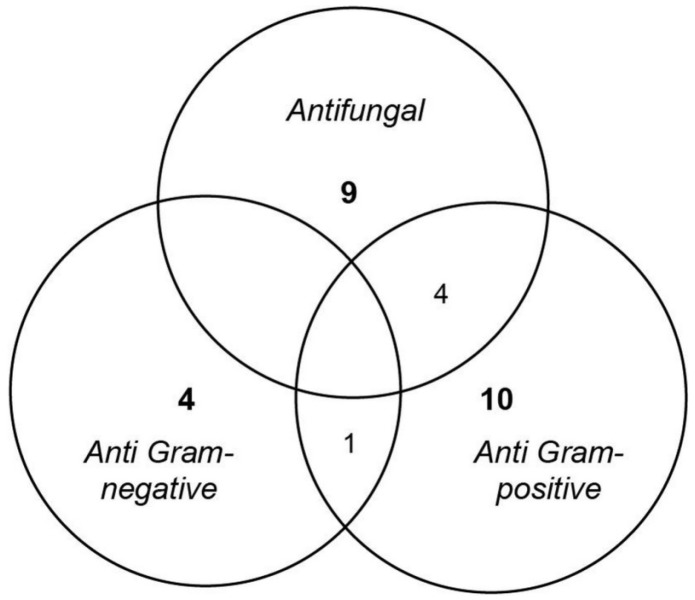
Venn diagram summarizing the spectrum of activity of the 28 antimicrobial producing deep subseafloor fungal isolates.

As clearly shown in the Venn diagram (Figure 2), most of the deep subseafloor fungal isolates produce narrow-spectrum antimicrobial compounds. Indeed, 82% of antimicrobial producing isolates are active against a specific category of microorganisms, *i.e.*, Gram-positive bacteria (10 isolates), fungi (nine isolates) or Gram-negative bacteria (four isolates). Only five isolates could be characterized as producers of a broader spectrum of activity with a mix of Gram-positive and fungal inhibition (*P. bialowiezense* CB_5, CB_7, CB_8 and *F. oxysporum* 4H1-P3-P3) or Gram-positive and Gram-negative inhibition (*A. terreus*1H3-S0-P1(1)).

This screening seems to exhibit a distribution pattern of antimicrobial activities with higher occurrence of antimicrobial producing fungal isolates at shallow sediment core depths (4–37 mbsf) compared to deeper layers (>100 mbsf) (Figure 1 and Figure S1). It is thus attractive to try glimpsing for any statistical correlations between antimicrobial potential and some environmental parameters, as depth or microbial richness.

### 2.2. Ecological Significance

**Figure 3:**
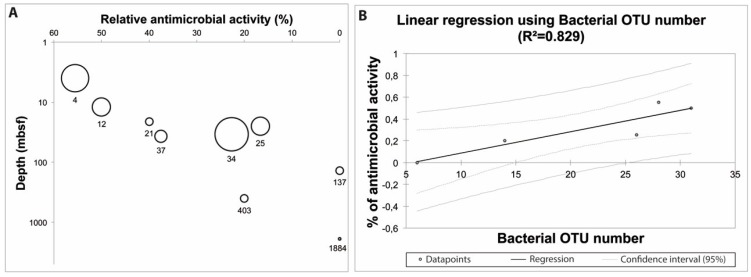
Antimicrobial activity along the sediment core. (**A**) Antimicrobial activity (%) expressed as a function of depth (size of the circles is directly linked to the number of screened fungal isolates, from one isolate (1884 mbsf) to 22 isolates (34 mbsf); (**B**) Relationship between antimicrobial activity (%) and bacterial Operational Taxonomic Unit (OTU) number (used as a proxy to estimate the complexity of the different sediment samples). The number of prokaryotic OTU was 28, 31, 26, 14 and 6 between 4–6 mbsf, 15–12 mbsf, 24–37 mbsf, 346–403 and 1827–1884 mbsf, respectively. The reader is referred to Ciobanu *et al.* [13].

Specific attention on the antimicrobial screening revealed a pattern of activity along the sediment core with a trend consisting in decreasing of the proportion of strains producing antimicrobial compounds when depth increases (Figure 3A). Indeed, relative occurrence of fungal isolates synthesizing bioactive compounds was higher at shallow depths compared to deeper layers.

Deep subseafloor sediments harbor a great reservoir of microbial cells, from 10^3^ to 10^8^ cells/cm^3^ [51,52] decreasing with depth [53]. Such a global trend was also obtained in the Canterbury basin sediment core with a significant decrease of bacterial and archaeal OTUs [13]. Linear regression was thus processed (Figure 3B) using diversity richness (in terms of Operational Taxonomic Units) at different depth layers, an exception being made for 765 mbsf depth, which was not used for diversity analyses [13]. Such analysis revealed a significant correlation between bacterial OTU number and deep subseafloor antimicrobial activity (*p*-value = 0.032), indicating that the more complex the microbiota is (NB: the highest OTU is observed in the shallow-layer sediment), the more the potential of deep subseafloor fungal isolates to synthesize antimicrobial compounds is important. From an ecological point of view, it seems to indicate that complex deep-sea ecosystems harboring a high diversity of microorganisms, with complex interactions to maintain/settle/colonize, can be allegorized as untapped chemical battlefields, as previously defined in other marine ecosystems [54,55,56].

### 2.3. Antimicrobial Compounds Production and Extraction

From the 28 deep subseafloor fungal isolates synthesizing antimicrobial compounds, a selection was performed according to different criteria as novelty, isolation depth and genetic potential based on a previous study [13]. Doing so, six strains namely, *Aspergillus fumigatus* 48X3-P3-P1(2), *Eurotium herbariorum* CB_33, *Penicillium bialowiezense* CB_7, *Penicillium* sp. CB_16, *Oidiodendron griseum* CB_36 and *Sarocladium* sp. 5H3-M3-P2 have been selected for deeper investigations to get insights into an optimized culture condition for an efficient production of bioactive metabolites. *Sistrotema brinkmanii* 5H1-P0-P5(1)bis was exluded because of its partial activity (Figure 1).

After extraction, antimicrobial activity was checked against the most sensitive target (see Figure 1). An exception was made for *Sarocladium* sp 5H3-M3-P2, antimicrobial activity was recovered and thus highlights the amphipathic chemical nature of the secreted bioactive compounds (Figure 2). Overall, as no antimicrobial activity was ever detected from culture in liquid medium Potato Dextrose Broth (PDB), semi-liquid (PDB complemented with 0.2% agar, (PDA 0.2%) or solid (PDB complemented with 1.7% agar, PDA 1.7%) medium were the best culture conditions to be used for an efficient antimicrobial production.

These results clearly indicate that specific culture conditions, *i.e.*, here liquid *versus* solid-state fermentation, trigger antimicrobial production. Interestingly, agar-based media seem to boost the production of antimicrobial compounds as no activity was ever detected in C18-SPE cell-free supernatant fractions (F10-90) of strains cultivated in PDB (data not shown). Interestingly, Bigelis *et al.* [57] have demonstrated that only solid-state fermentation allowed the fungal synthesis of antimicrobial compounds, albeit such organisms were able to grow in liquid media and thus support our findings on deep subseafloor marine fungi. As a result, solid state fermentation recently has received growing interest [58]. On the other hand, submerged fermentation have been extensively used as a large scale method to maximize the production of bioactive metabolites. We have assessed different kinds of fermentation: submerged fermentation (PDB and PDA 0.2%) and solid-sate fermentation (PDA 1.7%). No positive correlation was observed between the increasing agar concentration and the bioactivity. Culture conditions that stimulate the production of antimicrobial compounds thus appear strain-dependent, the different culture conditions leading to different metabolisms as desmonstrated in literature [59,60].

**Figure 4:**
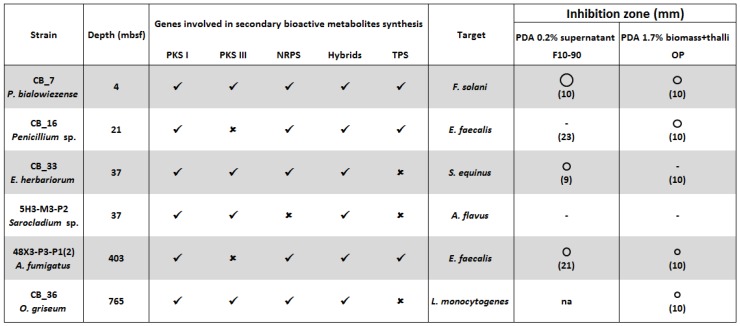
Antimicrobial activity after extraction. 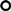: size of the inhibition zone 2, 3 and 5 mm. ✓ Presence, or ✗ absence of genes coding PKS I, PKS III, NRPS, PKS-NRPS and TPS [15]. F10-90: C18-SPE fraction eluted with acetonitrile 90%. OP: Organic phase. In bracket the concentration (mg/mL) of the crude extract. Na: Not assayed

The antimicrobial producing deep subseafloor fungal isolates harbor many genes involved in the biosynthesis of secondary metabolites (Figure 4). If this genetic pattern illustrates the biotechnological potential of the deep-sea fungal isolates, it does not allow to clearly identify the chemical nature of the bioactive compounds but rather to obtain hints. Here, polyketides, non ribosomal peptides and/or terpenes might be responsible for the detected antimicrobial activities. LC-MS/MS analyses coupled with a dereplication strategy will allow researchers to go deeper into the characterization of the deep subseafloor fungal chemodiversity.

## 3. Materials and Methods

### 3.1. Strains Collection

Marine fungal strains were isolated from a sediment core drilled in the Canterbury Basin on the eastern margin off New Zealand during the Integrated Ocean Drilling Program (IODP) Leg 317 Expedition [15]. All the isolated strains were identified, characterized and cryopreserved at −80 °C in the Université de Bretagne Occidentale Culture Collection (UBOCC) [61]. Here, 110 fungal isolates were chosen as an exhaustive strain sub-collection in order to select all representatives of the different taxonomic clusters isolated at different depths, from 4 to 1884 mbsf.

### 3.2. Cultivation Methods

Filamentous fungal isolates were cultivated on Potato Dextrose Agar (PDA) for 7 days at 25 °C. Then, isolates were subcultured onto PDA plates by central inoculation for 14 days at 25 °C. Spores suspension were calibrated at 1 × 10^7^ spores/mL and 500 µL were used to inoculate 50 mL liquid medium (PDB) or semi liquid (PDB + 0.2% Agar; PDA 0.2%). Solid medium (PDA) was centrally inoculated by dropping 10 µL of the spores suspension (1 × 10^7^ spores/mL). All media were incubated at 25 °C for 14 days. Broths were under rotary shaking (100 rpm) and plates were in static conditions.

Yeast isolates were first cultivated on Yeast extract Peptone Dextrose Agar (YPDA) for 2 days at 25 °C. Then, one colony was used to inoculate a 5 mL YPD Broth (YPDB) for 24 h at 25 °C with rotary shaking at 100 rpm. Finally 50 µL of this pre-culture was incubated in 5 mL of YPDB during 4 days at 25 °C and 100 rpm.

### 3.3. Antimicrobial Screening

Sixteen microbial targets, belonging to human or marine animal pathogenic bacteria and fungi were selected in different culture collections: (i) *Enterobacter aerogenes* CIP 6086, *Escherichia coli* ATCC 25922, *Klebsiella oxytoca* CIP 7932, *Pseudomonas aeruginosa* ATCC 27853, *Salmonella enterica* CIP 8297, *Vibrio parahaemolyticus* Ifremer 01/252 and *Yersinia ruckeri* ATCC 29473, representing the Gram-negative bacterial targets; (ii) *Enterococcus faecalis* CIP A 186, *Lactococcus garviae* ATCC 43921, *Listeria monocytogenes* SOR 100 *Staphylococcus aureus* ATCC 25923, *Streptococcus equinus* NRRL B-4268 representing the Gram-positive bacterial targets. Antimicrobial activity was also assessed against different filamentous fungi: *Aspergillus flavus* CBS 100927, *Exophiala dermatidis* UBOCC-A-113043, *Fusarium solani* CBS 181.29 and the yeast *Candida albicans* ATCC 2092. All of the target cells were cultivated according to the ATCC standards.

For antimicrobial assays, bacterial and yeast target cells were respectively included in Trypton Soy and in YPDA agar at 1 × 10^6^ cfu/mL, while, filamentous fungi spores were included at 1 × 10^5^ spores/mL in PDA.

Antimicrobial screenings were assessed using agar diffusion methods. After 4 days incubation for yeast, 5 µL of culture was inoculated into a well made in the target cell medium. While, after 14 days incubation for filamentous fungi a plug of culture was dropped off into a well made in the target cell medium. The antimicrobial activity was expressed as the radius of the inhibition zone in mm.

### 3.4. Extraction of Amphipathic Bioactive Compounds

Fungal isolates producing antimicrobial compounds were cultivated during 14 days in PDB, PDA 0.2% and PDA 1.7%. In order to extract amphipathic metabolites, cell-free supernatants (20 mL) from PDB and PDA 0.2%, were collected (centrifugation, 8000 *g*, 4 °C, 40 min), loaded and fractionated onto Solid Phase Extraction C-18 cartridge (SPE/C18 UPTI-clean, Interchim, France). After a first washing step performed with 10% acetonitrile +0.07% Trifluoroacetic Acid (TFA), the retained compounds were eluted using 90% acetonitrile +0.07% TFA in a fraction named F10-90. and finally freeze-dryed [62].

Biomass and solid culture media (PDA 1.7%) were collected crushed in liquid nitrogen and then extracted using Methanol/Chloroform/Water (4V/5V/1V; 1 g/10 mL) for 10 min at 100 rpm and finally centrifuged (4 °C, 8000 *g*, and 20 min). The aqueous phase was removed, and Organic Phase (OP) was dried under vacuum and solubilized in 20% acetonitrile (1 g of fresh mycelium/5 mL). All fractions of interest (F10-90 and OP) were freeze-dried and solubilized in 20% acetonitrile. Since the examined fractions arose from an extraction onto non polar phase and then were soluble in a more polar solvent (*i.e.*, 20% acetonitrile), they were considered as made of amphipathic compounds.

## 4. Conclusions

Our study revealed the antimicrobial properties of a unique deep subseafloor fungal collection obtained from a record-depth sediment core. A sub-collection of 110 fungal strains was screened for antimicrobial activities against 16 Gram-negative, Gram-positive and fungal targets. A relatively high proportion of bioactivity was obtained as 33% of filamentous fungi inhibit the growth of at least one microbial target. Antimicrobial activities were mainly directed towards Gram-positive bacteria and fungi. To the best of our knowledge, this is the first screening to deal with deep subseafloor fungi. Here, the high proportion of fungi producing antimicrobial compounds coupled with the evidence that deep subseafloor fungi possess a range of adaptations to cope with numerous biological, physical and chemical stressors clearly highlights such organisms as an untapped reservoir to investigate for novel molecules of biotechnological interest. Complementary analyses will now been focused on the fractionation, dereplication and identification of molecules in search of putatively novel antimicrobial compounds.

## Supplementary Materials

The following are available online at http://www.mdpi.com/1660-3397/14/3/50/s1, Figure S1: Antimicrobial screening of 110 deep subseafloor fungi.
