# Supplementary figures and images for: Deep Subseafloor Fungi as an Untapped Reservoir of Amphipathic Antimicrobial Compounds
Source: Mar Drugs. 2016 Mar 10;14(3):50. doi: 10.3390/md14030050 (PMC4820304; doi:10.3390/md14030050)

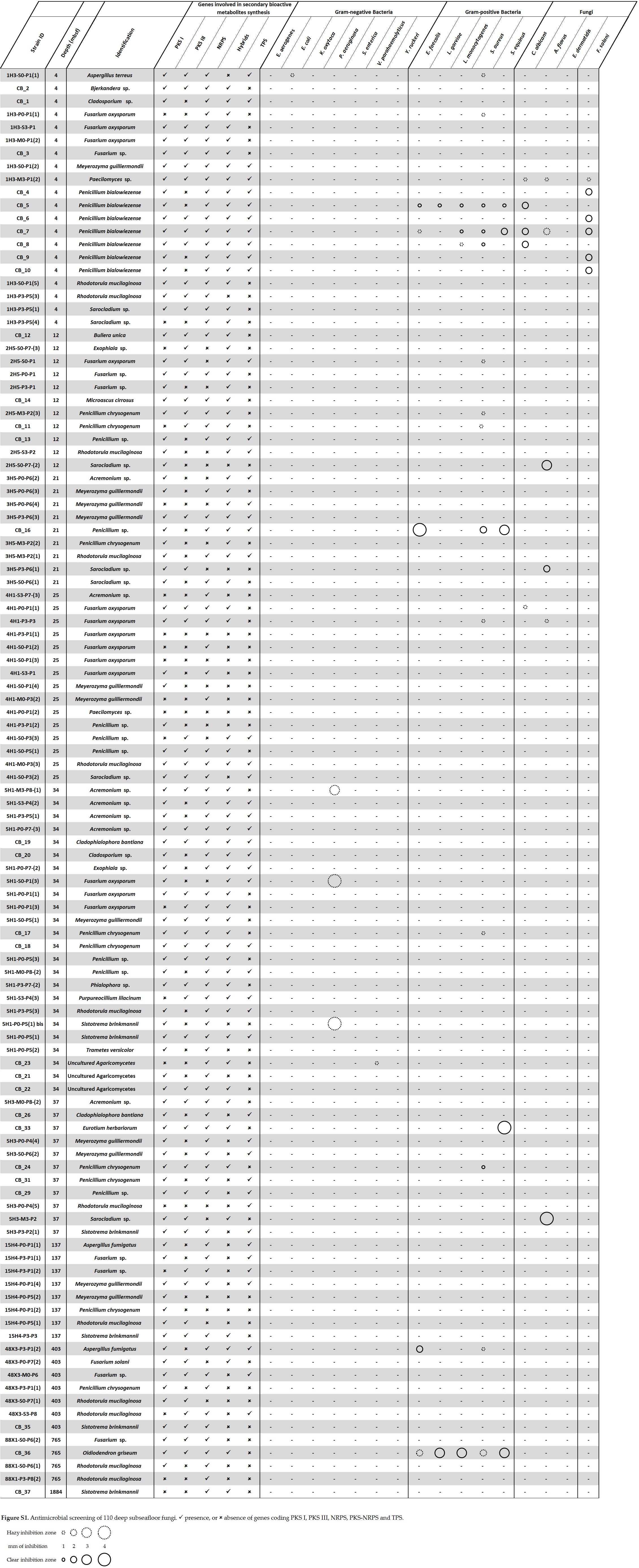

Supplement: Supplementary file 1 [file marinedrugs-14-00050-s001.jpg]
